# Association Study of the M132L Single Nucleotide Polymorphism With Susceptibility to Chronic Wasting Disease in Korean Elk: A Meta-Analysis

**DOI:** 10.3389/fvets.2021.804325

**Published:** 2022-01-14

**Authors:** In-Soon Roh, Yong-Chan Kim, Sae-Young Won, Kyung-Je Park, Hoo-Chang Park, Ji-Yong Hwang, Hae-Eun Kang, Hyun-Joo Sohn, Byung-Hoon Jeong

**Affiliations:** ^1^Reference Laboratory for Chronic Wasting Disease (CWD), Foreign Animal Disease Division, Animal and Plant Quarantine Agency, Gimcheon, South Korea; ^2^Korea Zoonosis Research Institute, Jeonbuk National University, Iksan, South Korea; ^3^Department of Bioactive Material Sciences and Institute for Molecular Biology and Genetics, Jeonbuk National University, Jeonju, South Korea

**Keywords:** Elk (*Cervus canadensis*), prion, CWD, PRNP, M132L, polymorphism, SNP, meta-analysis

## Abstract

Chronic wasting disease (CWD) is a deleterious brain proteinopathy caused by a pathogenic form of prion protein (PrP^Sc^), which is converted from a benign form of prion protein (PrP^C^) encoded by the prion protein gene (*PRNP*). In elk, the M132L single nucleotide polymorphism (SNP) of the *PRNP* gene likely plays a pivotal role in susceptibility to CWD. However, the association of the M132L SNP with susceptibility to CWD has not been evaluated in Korean elk to date. To estimate the association of the M132L SNP with susceptibility to CWD in Korean elk, we investigated the genotype and allele frequencies of the M132L SNP by amplicon sequencing and performed association analysis between CWD-positive and CWD-negative elk. In addition, we performed a meta-analysis to evaluate the association between the M132L SNP and susceptibility to CWD in quantitatively synthesized elk populations. Furthermore, we estimated the effect of the M132L SNP on elk PrP using *in silico* programs, including PolyPhen-2, PROVEAN, AMYCO and Swiss-PdbViewer. We did not identify a significant association between the M132L SNP of *PRNP* and susceptibility to CWD in Korean elk. The meta-analysis also did not identify a strong association between the M132L SNP of *PRNP* and susceptibility to CWD in quantitatively synthesized elk populations. Furthermore, we did not observe significant changes in structure, amyloid propensity or electrostatic potential based on the M132L SNP in elk PrP. To the best of our knowledge, this was the first report of an association analysis and meta-analysis in Korean elk and quantitatively synthesized elk populations, respectively.

## Introduction

Prion diseases are infectious brain proteinopathies caused by a pathogenic form of prion protein (PrP^Sc^) converted from an endogenous form of prion protein (PrP^C^) and are classified into several types in a wide range of mammalian hosts: Creutzfeldt–Jakob disease (CJD), fatal familial insomnia (FFI) and Gerstmann-Sträussler-Scheinker syndrome (GSS) in humans; scrapie in sheep and goats; bovine spongiform encephalopathy (BSE) in cattle; and chronic wasting disease (CWD) in the Cervidae family (1–7). After CWD was first reported in mule deer in the USA in the 1967, CWD has been reported globally, including in North America, South Korea and Scandinavia, in variable cervid species, such as white-tailed deer, elk, moose, red deer and reindeer (8–11). To date, CWD is a fatal and incurable disorder, and its exact origin has not been elucidated to date.

Prion protein (PrP), which is encoded by the prion protein gene (*PRNP*), is the template of ${\text{PrP}}_{,}^{\text{Sc}}$ and *PRNP* gene variations are the primary factor in determining susceptibility to CWD (8, 12). In elk, the M132L single nucleotide polymorphism (SNP) of the *PRNP* gene, which is equivalent to codon 129 in the human *PRNP* gene, plays a pivotal role in susceptibility to CWD. A previous case-control study reported that the M132L SNP of the *PRNP* gene is associated with vulnerability to CWD in elk in the USA (13). The M132 allele was more frequently observed in CWD-positive elk, and the L132 allele was not observed in CWD-positive elk. In addition, CWD-inoculated elk PrP transgenic mice with the L132 allele showed resistance to CWD compared to elk PrP transgenic mice with M132 allele (14). Furthermore, experimentally CWD-infected elk carrying the 132LL genotype showed extended survival times compared to elk carrying the 132MM and 132ML genotypes (15). However, another case-control study did not report the association between the M132L SNP and susceptibility to CWD in the USA (16). In addition, an *in vitro* conversion assay using real-time quaking-induced conversion reactions (RT-QuIC) showed that regardless of the genotype of *PRNP* codon 132, the conversion efficiency of CWD was high when the genotype of this codon was matched (15). Thus, the effect of the *PRNP* M132L SNP on the risk of CWD remains controversial.

In Korea, >12,000 elk are bred to obtain meat and antlers as food and medicine, respectively (https://www.mafra.go.kr). Korean elk were originally imported from North America, and CWD in Korean elk has been reported sporadically (17). Given that CWD is a highly infectious prion disease and can be transmitted by peripheral body fluids, including urine, tears and saliva, it is very important to breed elk that are genetically resistant to CWD for preemptive control of CWD (18). However, the M132L SNP, one of the important genetic factors of CWD, has not been investigated in Korean elk to date.

To evaluate the association of the *PRNP* M132L SNP with susceptibility to CWD in Korean elk, we investigated the genotype and allele frequencies of this SNP by amplicon sequencing and performed association analysis between CWD-positive and CWD-negative elk. In addition, we performed an integrative evaluation of the association between the *PRNP* M132L SNP and susceptibility to CWD in quantitatively synthesized elk populations by meta-analysis. Furthermore, we estimated the effect of the M132L SNP on elk PrP using *in silico* programs, including PolyPhen-2, PROVEAN, AMYCO, and Swiss-PdbViewer (19–21).

## Materials and Methods

### Ethics Statements

All experimental procedures were approved according to the recommendations of the Institutional Animal Care and Use Committee of Jeonbuk National University (IACUC Number: JBNU-2019-0076). All experiments were performed in accordance with the Korea Experimental Animal Protection Act.

### Subjects

A total of 253 brain samples were collected from animal farms in the Republic of Korea where CWD has occurred in elk. CWD tests were performed on all brain samples by the Animal and Plant Quarantine Agency (APQA) in the Republic of Korea using the HerdChek BSE-Scrapie Antigen Kit (IDEXX, USA) and western blot analysis. In 253 elk, 49 elk were infected with CWD.

### Genomic DNA Extraction

Genomic DNA was eluted from 20 mg brain tissue using a QIAamp DNA Mini Kit (Qiagen, USA) following the manufacturer's instructions.

### Genetic Analysis of the M132L SNP of the Elk *PRNP* Gene

The elk *PRNP* gene (accession number: FJ590751.1) was amplified from the genomic DNA using sense and antisense gene-specific primers. The sequences of the primers were as follows: PRNP-F (ATGGTGAAAAGCCACATAGGC) and PRNP-R (ACACTTGCCCCTCTTTGGTA). Polymerase chain reaction (PCR) was performed using GoTaq® DNA Polymerase (Promega, Fitchburg, Wisconsin, USA). The PCR mixture contained 20 pmol of each primer, 5 μl of 10× *Taq* DNA polymerase buffer, 1 μl of 10 mM dNTP mixture and 2.5 units of *Taq* DNA polymerase. The PCR conditions for the PRNP-F and PRNP-R primers were 95°C for 2 min for denaturation; 35 cycles of 94°C for 45 s, 59°C for 45 s, and 72°C for 1 min 30 s; 1 cycle of 72°C for 10 min for extension. PCR was performed using an S-1000 Thermal Cycler (Bio–Rad, Hercules, California, USA). The PCR products were purified using a PCR Purification Kit (Thermo Fisher Scientific, Bridgewater, New Jersey, USA) and sequenced using an ABI 3730 automatic sequencer (ABI, Foster City, California, USA). Sequencing results were read by Finch TV software (Geospiza Inc., Seattle, USA), and M132L SNP genotyping of the elk *PRNP* gene was performed.

### Literature Search

A literature search was performed using the PubMed database to identify studies regarding the M132L SNP of the *PRNP* gene from CWD-affected elk. The following search terms were used: “elk,” “*PRNP*,” “prion,” “CWD,” or “chronic wasting disease” combined with “SNP,” “polymorphism,” or “susceptibility” (the last search update was performed on July 30, 2021). We also supplemented our search results by checking the reference lists of all the relevant studies, including original articles and reviews. Eligible studies met the following inclusion criteria: (1) regarding the association between the *PRNP* M132L SNP and CWD; (2) case-control study; (3) genetic information on the M132L SNP of CWD-affected elk; (4) full text; and (5) published in English. The exclusion criteria were as follows: (1) case reports and (2) insufficient genotype data.

### Meta-Analysis

The strength of the integrated association between the M132L SNP of the *PRNP* gene and susceptibility to CWD was estimated by meta-analysis. The pooled odds ratios with 95% confidence intervals were estimated using additive (M vs. L), recessive (MM vs. ML+LL), dominant (MM+ML vs. LL), and overdominant (ML vs. MM+LL) genetic models and homozygote (MM vs. LL) and heterozygote (MM vs. ML and ML vs. LL) comparisons. Heterogeneity was calculated based on the *P*-value and *I*^2^-value. Fixed effect models were selected to calculate the pooled odds ratios according to the value of the *I*^2^-test. Publication bias was calculated using Egger's weighted regression methods. Meta-analysis was performed using the meta package of the R program (https://www.r-project.org/).

### *In silico* Analysis of the Impact of the M132L SNP on Elk PrP

PolyPhen-2 (http://genetics.bwh.harvard.edu/pph2/index.shtml) and PROVEAN (http://provean.jcvi.org/seq_submit.php) were used to predict the impact of the M132L SNP. PolyPhen-2 was predicted based on several characteristics of the target protein, including the sequence, structural and phylogenetic information characterizing the variation. PolyPhen-2 yields the following estimation results, including benign, possibly damaging, or probably damaging, based on scores ranging from 0.0 to 1.0. The PROVEAN scores were calculated based on clustering basic local alignment search tool (BLAST) hits according to the homologs collected from a database (the NCBI nr database). PROVEAN scores were classified as follows: below −2.5 indicates “deleterious,” and above−2.5 indicates “neutral.” AMYCO analysis was performed to estimate the effect of the M132L SNP on the aggregation propensity of elk PrP based on the combined score of the pWALTZ and PAPA algorithms. An AMYCO score <0.45 indicates low amyloid properties.

### 3D Structure and Electrostatic Potential Analyses

The nuclear magnetic resonance (NMR) structure of elk PrP was obtained from the RCSB Protein Data Bank (PDB ID: 1XYW). The electrostatic potential according to the M132L SNP was also analyzed using the Swiss-PdbViewer 4.1 program (https://spdbv.vital-it.ch/).

### Statistical Analysis

Statistical analyses were performed using SAS version 9.4 (SAS Institute Inc., USA). The differences in genotype and allele frequencies of the *PRNP* gene between CWD-negative and CWD-positive animals were compared using the χ^2^-test and Fisher's exact test. The Hardy-Weinberg equilibrium (HWE) test was performed using Haploview version 4.2 (Broad Institute, Cambridge, MA, USA).

## Results

### No Association Between the M132L SNP of the Elk *PRNP* Gene and Susceptibility to CWD in the Republic of Korea

To investigate the association between the M132L SNP of the elk *PRNP* gene and susceptibility to CWD, we performed amplicon sequencing and genotyping in 253 elk, including 204 CWD-negative elk and 49 CWD-positive elk. Detailed information on genotype and allele frequencies is described in Table 1. Notably, the genotype and allele frequencies of the M132L SNP were not significantly different between CWD-negative and CWD-positive elk in the Republic of Korea (Table 1).

**Table 1:** Comparison of genotype and allele frequencies of the M132L single nucleotide polymorphism (SNP) in the prion protein gene (*PRNP*) between chronic wasting disease (CWD)-affected elks and matched healthy elks.

**Authors**	**Year**	**Country**		**Total, *n***	**Genotype frequencies**, ***n***			***P*-value^**a**^**	**Allele frequencies**, ***n***		***P*-value^**b**^**	**HWE**
					**MM**	**ML**	**LL**		**M**	**L**		
O'Rourke et al. (13)	1999	USA	CTL	344	230	102	12	**0.0312**	562	126	**0.0084**	0.8677
			CWD	43	37	6	0		80	6		0.6229
Perucchini et al. (16)	2008	USA	CTL	248	162	80	6	0.6927	404	92	0.4294	0.2872
			CWD	94	66	26	2		158	30		0.7621
In this study	2021	South Korea	CTL	204	144	49	11	0.9326	337	71	0.8000	0.0188
			CWD	49	35	12	2		82	16		0.4681

*The number of genotype frequencies in each column indicates the number of genotypes. The number reported in each “Allele frequencies” column indicates the number of alleles. The number “Total, n” indicates the number of animals. Bold text indicates statistically significant results (P < 0.05). CTL, matched healthy elks; CWD, CWD-affected elk; HWE, Hardy-Weinberg equilibrium; P-value^a^: Based on the comparison of genotype frequencies between healthy controls and CWD-affected animals; P-value^b^: Based on the comparison of allele frequencies between healthy controls and CWD-affected animals*.

### Evaluation of the Association Between the M132L SNP of the Elk *PRNP* Gene and Susceptibility to CWD by Meta-Analysis

We searched 210 research articles following the search terms “*PRNP*,” “prion,” “CWD,” or “chronic wasting disease” combined with “SNP” or “polymorphism” or “susceptibility” (the last search update was performed on July 30, 2021) in PubMed. After excluding duplicate and irrelevant articles, a total of 2 eligible studies were extracted from the PubMed database based on our inclusion and exclusion criteria.

To identify an association between the M132L SNP of the elk *PRNP* gene and susceptibility to CWD in each previous study, we performed an association analysis in 2 previous studies performed in the USA (Table 1). Notably, the genotype and allele frequencies of the M132L SNP of the elk *PRNP* gene exhibited a strong association (*P* < 0.05) with susceptibility to CWD in a study by O'Rourke et al. (13) (Table 1). However, the genotype and allele frequencies of the M132L SNP of the elk *PRNP* gene did not show a strong association with vulnerability to CWD in a study by Perucchini et al. (16).

**Figure 1:**
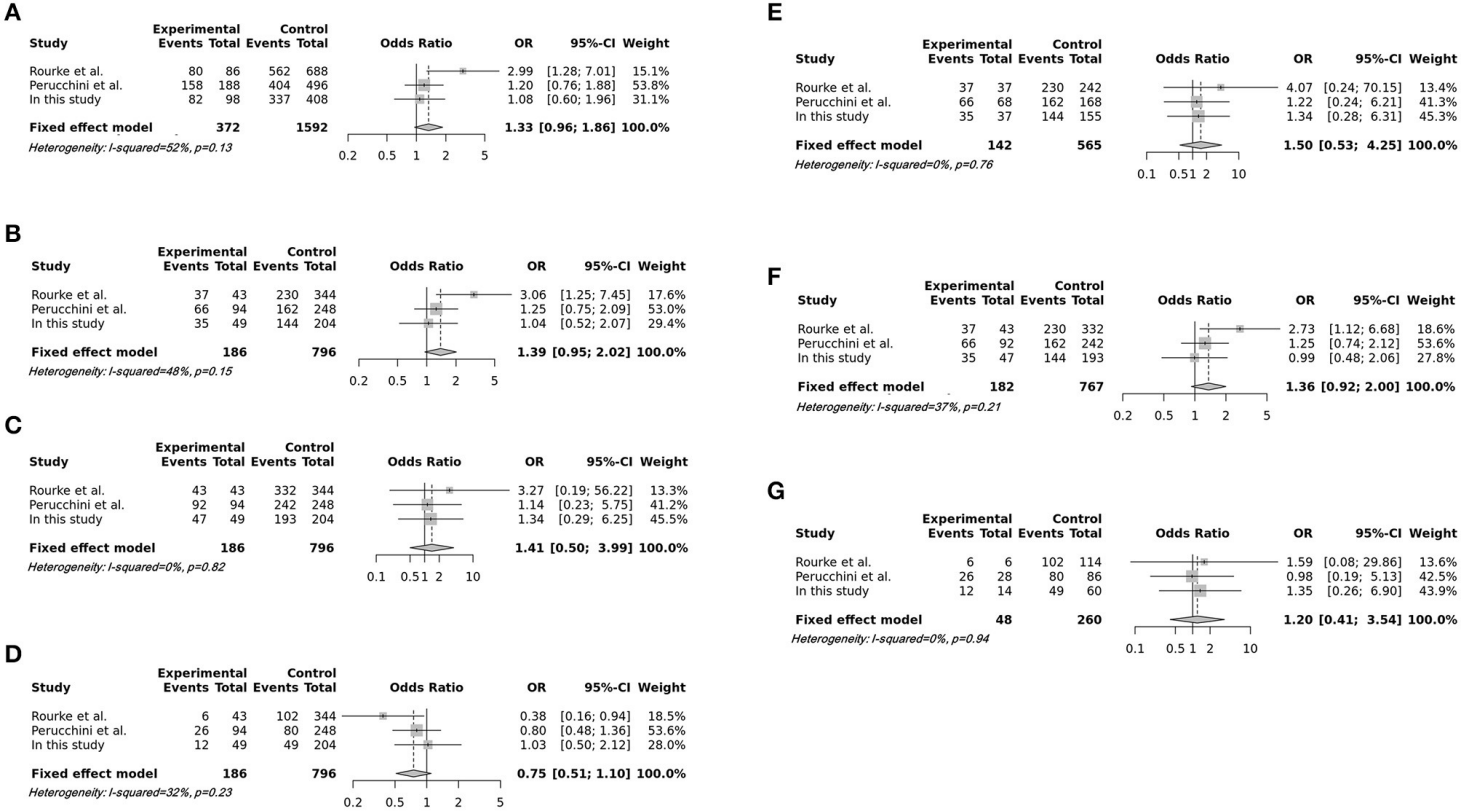
Forest plots of the association between the M132L single nucleotide polymorphism (SNP) of the elk prion protein gene (*PRNP*) and susceptibility to chronic wasting disease (CWD). **(A)** Forest plots of the association between the M132L SNP of the elk *PRNP* gene and susceptibility to CWD in the additive model (M vs. L). **(B)** Forest plots of the association between the M132L SNP of the elk *PRNP* gene and susceptibility to CWD in the recessive model (MM vs. ML+LL). **(C)** Forest plots of the association between the M132L SNP of the elk *PRNP* gene and susceptibility to CWD in the dominant model (MM+ML vs. LL). **(D)** Forest plots of the association between the M132L SNP of the elk *PRNP* gene and susceptibility to CWD in the overdominant model (ML vs. MM+LL). **(E)** Forest plots of the association between the M132L SNP of the elk *PRNP* gene and susceptibility to CWD in the homozygote comparison (MM vs. LL). **(F)** Forest plots of the association between the M132L SNP of the elk *PRNP* gene and susceptibility to CWD in the heterozygote comparison (MM vs. ML). **(G)** Forest plots of the association between the M132L SNP of the elk *PRNP* gene and susceptibility to CWD in the heterozygote comparison (ML vs. LL).

In total, 796 CWD-negative elk and 186 CWD-positive elk were included in the meta-analysis. The integrated association was evaluated by pooled odds ratios with 95% confident intervals using additive, recessive, dominant, and overdominant genetic models and homozygote and heterozygote comparisons. Heterogeneity was not observed (*I*^2^ <50%). Then, we used a fixed-effect model for the meta-analysis (Table 2). Notably, all meta-analyses using several genetic models and homozygote and heterozygote comparisons showed no association between the risk of CWD and the M132L SNP of the elk *PRNP* gene (Figure 1, Table 2). To examine potential publication bias, Egger's tests were performed, and publication bias was not observed in this meta-analysis (*P* > 0.1).

**Table 2:** Meta-analysis of the association between the M132L SNP of the *PRNP* gene and susceptibility to CWD.

**Genetic model**	**Association test**			**Heterogeneity**			**Publication bias**
	**Odds ratio**	**95% confidence interval**	***P*-value**	**Model**	***P*-value**	***I* ^2^**	**Egger's test *P*-value**
Additive model (M vs. L)	1.3325	[0.9570; 1.8553]	0.6246	Fixed	0.13	0.52	0.3852
Recessive model (MM vs. ML+LL)	1.3873	[0.9546; 2.0161]	0.6029	Fixed	0.15	0.48	0.4978
Dominant model (MM+ML vs. LL)	1.4120	[0.4998; 3.9891]	1	Fixed	0.82	0.00	0.1956
Overdominant model (ML vs. MM+LL)	0.7506	[0.5113; 1.1019]	1	Fixed	0.23	0.32	0.6099
MM vs. LL	1.4962	[0.5266; 4.2505]	1	Fixed	0.76	0.00	0.1181
MM vs. ML	1.3585	[0.9238; 1.9978]	0.8363	Fixed	0.21	0.37	0.5972
ML vs. LL	1.2004	[0.4067; 3.5433]	1	Fixed	0.94	0.00	0.6146

### *In silico* Evaluation of the Effect of the M132L SNP on Elk PrP

**Figure 2:**
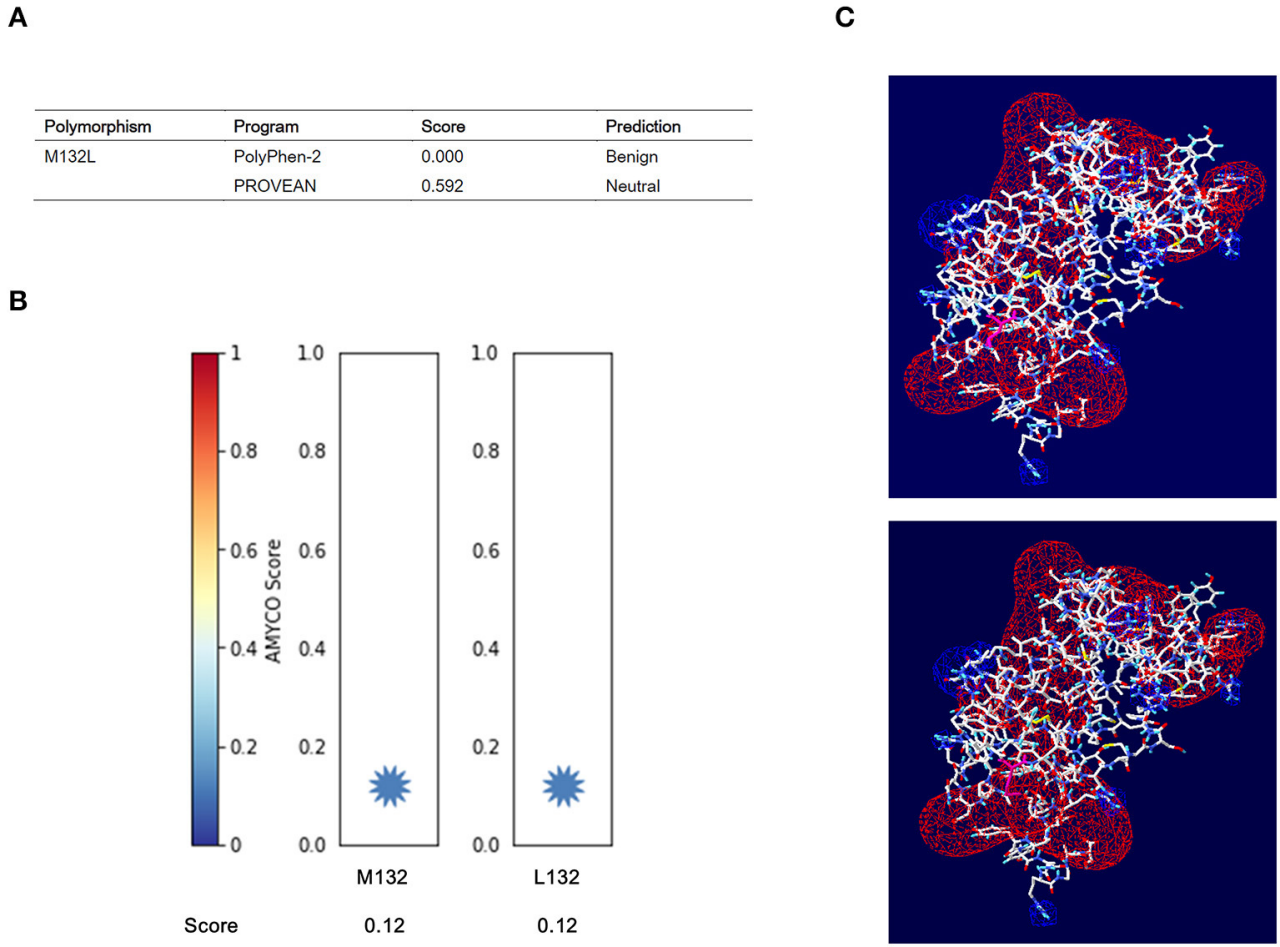
*In silico* evaluation of the effect of the M132L SNP on elk prion protein (PrP). **(A)** Prediction of the functional effect of the M132L SNP on elk PrP by PolyPhen-2 and PROVEAN. **(B)** Prediction of the aggregation propensity of elk PrP according to alleles at codon 132 using AMYCO. **(C)** Electrostatic potential and 3D structure analysis of elk PrP according to the M132L SNP. Upper panel: electrostatic potential and 3D structure of elk PrP with the M132 allele. Lower panel: electrostatic potential and 3D structure of elk PrP with the L132 allele. Positive potentials are drawn in blue. Negative potentials are noted in red.

We estimated the impact of the M132L SNP of the *PRNP* gene on elk PrP using PolyPhen-2 and PROVEAN. Detailed scores predicted by the two programs are described in Figure 2A. In brief, the M132L SNP was estimated to be “benign” and “neutral” by PolyPhen-2 and PROVEAN, respectively. In addition, elk PrP with the M132 allele showed an amyloid propensity similar to that of elk PrP with the 132L allele (Figure 2B). We evaluated the electrostatic potential and 3D structure analysis according to the M132L SNP. Notably, we did not find significant changes in the electrostatic potential and 3D structure of elk PrP according to the M132L SNP (Figure 2C).

## Discussion

In the present study, the M132L SNP of the *PRNP* gene was not associated with susceptibility to CWD in Korean elk (Table 1). Previous studies have reported that the genotype and allele frequencies of genetic polymorphisms of the *PRNP* gene in the Cervidae family can vary according to geographic areas of habitat (13, 16, 22). In addition, all Korean elk were raised on farms. Thus, the indigenous genetic features of Korean elk may be caused by geographically isolated habitats and breeding modes. In the present study, information on the age and sex of Korean elk was not available. Further analysis stratified by age and sex is highly desirable in the future.

We also integrated and evaluated the association of the M132L SNP of the *PRNP* gene with susceptibility to CWD in quantitatively synthesized elk populations by meta-analysis. Notably, we did not observe an association of the M132L SNP of this gene with susceptibility to CWD in any genetic models of meta-analyses (Table 2, Figure 1). Although previous experimental infections in elk and elk PrP transgenic mice showed the protective effect of the L132 allele, these results did not correspond with that of the present meta-analysis (14). In human prion diseases, the M129V SNP of the human *PRNP* gene plays a pivotal role in susceptibility to sporadic CJD (23, 24). However, the M129V SNP is not a solitary factor in the vulnerability of sporadic CJD. In the Asian population, the E219K SNP of the *PRNP* gene confers resistance to CJD (25–28). Although the overrepresented M129 allele of the *PRNP* gene was observed in Asian populations (>90%), including Korea and Japan, the incidence of sporadic CJD is similar and/or low compared to European populations, which have a relatively low frequency of the M129 allele (<70%). In addition, the G127V SNP of the *PRNP* gene was reported in Kuru-unaffected Papua New Guinean, and a follow-up study using transgenic mice carrying human PrP with the 127V allele confirmed that the V127 allele conferred potent resistance to several human prion diseases (29). Thus, given that the full amino acid sequences of the elk PrP of inoculation agents and recipient animals were not available in previous studies, further detailed studies applying other polymorphisms of elk PrP are highly desirable. With the exception of the M132L SNP of the elk *PRNP* gene, an association test for the entire amino acid sequence of elk PrP has not been performed. This association study is highly desirable in the future.

We also estimated the effect of the M132L SNP on elk PrP by *in silico* programs and did not find significant changes in structure, aggregation propensity or electrostatic potential according to the M132L SNP (Figure 2). In previous studies, human PrP with the 129M allele also showed similar characteristics to human PrP with the 129V allele. The protective effect of the 129V allele is thought to be associated with the inhibition of homotypic protein–protein interactions (28). In addition, a recent study using an *in vitro* conversion assay by RT-QuIC reported that conversion efficiency primarily depends on the correspondence of genotype at *PRNP* codon 132 of agent and recipient recombinant protein (15). However, our *in silico* analysis and previous *in vitro* and *in vivo* analyses did not reflect haplotypes of the elk *PRNP* gene, and further investigation of genetic polymorphisms and haplotypes of the elk *PRNP* gene is needed in the future.

## Conclusion

We investigated the M132L SNP of the elk *PRNP* gene in Korean elk and performed association analysis between CWD-positive and CWD-negative elk for the first time. We did not observe a significant association between the M132L SNP of the *PRNP* gene and susceptibility to CWD in Korean elk. The meta-analysis also did not identify a strong association between the M132L SNP and susceptibility to CWD in quantitatively synthesized elk populations. Furthermore, we did not find significant changes in structure, amyloid propensity or electrostatic potential according to the M132L SNP in elk PrP using *in silico* analyses. To the best of our knowledge, this was the first report of an association analysis and meta-analysis in Korean elk and quantitatively synthesized elk populations, respectively.

## Data Availability Statement

The datasets presented in this study can be found in online repositories. The names of the repository/repositories and accession number(s) can be found below: GenBank (Accession no. FJ590751.1).

## Ethics Statement

The animal study was reviewed and approved by Institutional Animal Care and Use Committee of Jeonbuk National University.

## Author Contributions

I-SR, Y-CK, H-JS, and B-HJ conceived, designed the experiment, and wrote the paper. I-SR, Y-CK, S-YW, K-JP, H-CP, and J-YH performed the experiments. Y-CK, S-YW, H-EK, and B-HJ analyzed the data. All authors have read and approved the final manuscript.

## Funding

This research was supported by the Basic Science Research Program and National Research Foundation (NRF) of Korea funded by the Ministry of Education (2017R1A6A1A03015876, 2021R1A6A3A010864), National Research Foundation of Korea (NRF) by the Korea government (2021R1A2C1013213), Ministry for Agriculture, Food and Rural Affairs (B-1543085-21-22-01), BK21 Plus Program in Department of Bioactive Material Sciences to Y-CK, and NRF (National Research Foundation of Korea) Grant funded by the Korean Government (NRF-2019-Fostering Core Leaders of the Future Basic Science Program/Global Ph.D Fellowship Program).

## Conflict of Interest

The authors declare that the research was conducted in the absence of any commercial or financial relationships that could be construed as a potential conflict of interest.

## Publisher's Note

All claims expressed in this article are solely those of the authors and do not necessarily represent those of their affiliated organizations, or those of the publisher, the editors and the reviewers. Any product that may be evaluated in this article, or claim that may be made by its manufacturer, is not guaranteed or endorsed by the publisher.
